# Functional and quality of life outcome after non-operatively managed proximal humeral fractures

**DOI:** 10.1007/s10195-017-0468-5

**Published:** 2017-08-22

**Authors:** Ronnart N. Kruithof, Henk A. Formijne Jonkers, Denise J. C. van der Ven, Ger D. J. van Olden, Tim K. Timmers

**Affiliations:** Department of Surgery, Meander Medical Center Amersfoort, P.O.-box 1502, 3800 BM Amersfoort, The Netherlands

**Keywords:** Fracture, Proximal humerus, Conservative treatment, Functional outcome, Quality of life, DASH, EQ-5D

## Abstract

**Background:**

Fractures of the proximal humerus are common and most often treated non-operatively. However, long-term follow-up studies focusing on functional results and quality of life in patients after this type of fracture are scarce. The primary aim of this study is to report the long-term functional and quality of life outcome in patients with a proximal humeral fracture.

**Materials and methods:**

A retrospective analysis of all consecutive patients undergoing non-operative treatment for a proximal humeral fracture in a level 2 trauma centre between January 2000 and December 2013 was performed. A database consisting of all relevant demographic, patient and fracture characteristics was created. Subsequently, a questionnaire containing the DASH (Disabilities of the Arm, Shoulder and Hand) score, EuroQol-5D (EQ-5D), VAS (visual analogue scale) score, and subjective questions was sent to all patients.

**Results:**

A total of 410 patients (65 male, 345 female) were included for analyses. Average follow-up was 90 ± 48 months. DASH-scores <15 were considered as good. A median DASH-score of 6.67 [0.83–22.50] was found. A significant lower DASH-score was seen in patients under the age of 65 compared to older patients (*p* < 0.001). In comparison to an age-matched general Dutch population, Health related Quality of Life (HrQoL) on the EQ-us was not significantly worse in our study population (difference 0.02). Strong (negative) correlation was found between DASH-score and VAS-score, and DASH-score and HrQoL, respectively *ρ* = −0.534 and *ρ* = −0.787.

**Conclusion:**

Long-term functional and quality of life outcomes are good in most patients after proximal humeral fractures, but negatively correlated to each other.

**Level of evidence:**

Level III.

## Introduction

Proximal humeral fractures are frequently seen on emergency departments and encompass around 5% of all fractures. In the US an annual incidence of 60 per 100,000 persons per year has been reported [16]. These fractures are unipolar distributed by age, therefore, incidences up to 260 per 100,000 persons per year are described in woman between 80 and 89 years of age [4]. Mostly, they tend to occur in mobile elderly woman as a consequence of decreased bone density mass. Only fractures of the (distal) radius and the hip are more frequently seen, making it the third most common fracture in this particular group [4, 22, 23, 32]. A 50% increase in incidence of this fracture is expected due to the ageing of the population [16, 26, 34].

Of all fractures of the proximal humerus, about 85–90% are considered suitable for non-operative treatment: Immobilization for one or two weeks, followed by phsysiotherapy [19]. In these cases, fractures usually only show minor displacement, as well as little angulations, healing uneventfully in the future [31]. Indications for surgical treatment are usually based on factors like age, number of fragments, degree of displacement, baseline functional status of the patient, hand dominance, and surgical experience of the treating physician [36]. Nevertheless, no consensus has been reached yet, whether to perform conservative or surgical treatment after a proximal humeral fracture in particular cases.

Since the bones affected by a proximal humeral fractures are part of a joint, they can be classified as articular fractures, and sometimes even intra-articular. Therefore, the question arises if arthroses will occur. Therefore, long-term follow-up is needed. Formerly, until today fracture consolidation was the most important goal in therapy. Nowadays, functional outcome and quality of life (QoL) are increasingly important [38].

Most previous published studies report short-term results up to 24 months after non-operative treatment or compare different treatment modalities [8, 10, 30, 31, 35, 38]. Long-term results focusing on patient reported outcomes are lacking. Therefore, purpose of this large cohort study was to report long-term functional and quality of life outcomes after conservative treatment of fractures of the proximal humerus.

## Materials and methods

### Study design

All successive patients, who underwent non-operative treatment after a proximal humeral fracture between the first of January 2000 and the 31st of December 2013 at our trauma-surgical department were cross-sectionally analyzed. Our hospital is categorized as a Level 2 trauma centre in this region, where over 40,000 patient are treated annually at the accident and emergency department. Our study was approved by our local medical ethics committee and is also in line with the policy statement, declared in the Declaration of Helsinki (1975), as revised in 1983.

### Patient selection

All patients presenting with a fracture of the proximal humerus at the emergency department in the aforementioned timeframe, were included. Exclusion criteria were (1) an age under 18 years old at presentation, (2) patients who had surgical intervention after non-operative treatment was started, (3) patients who suffered from severe cognitive impairment and, therefore, were unable to fill out the questionnaires, (4) patients who were deceased at the time of this study, (5) unavailable contact information, and (6) patients who did not provide informed consent for participation.

### Data extraction methods

Relevant patient characteristics as well as demographic data and medical histories were gathered from the patient medical records, together with characteristics of the fracture and follow-up data.

Outcome measures were retrieved by the Disabilities of the Arm, Shoulder and Hand (DASH) questionnaire and the Euro-Qol 5Dimensions (EQ-5D) questionnaire. An additional questionnaire to retrieve information on dominance, occupation, and pre-injury functioning was also added. This questionnaire has not been validated. The three questionnaires were sent by regular post. If response failed to appear, patients were reminded by telephone contact once.

### Patient population

**Fig. 1:**
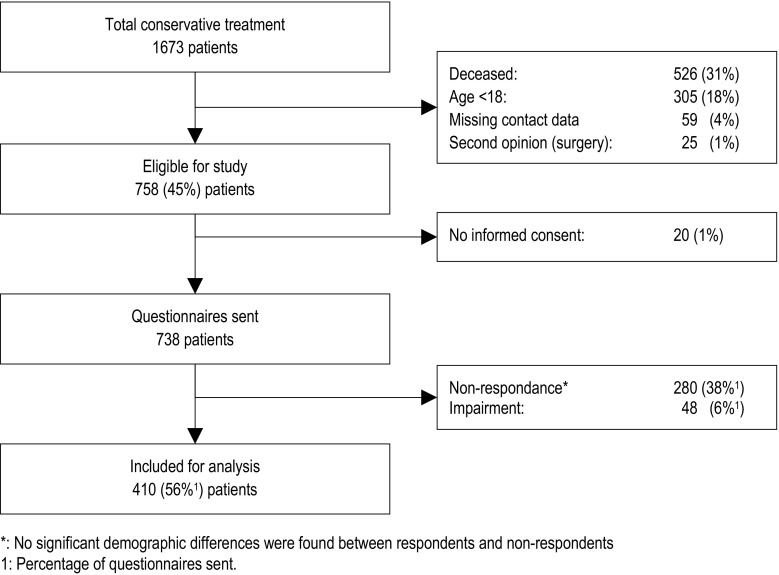
Study population flow chart

After application of the inclusion criteria, a total of 1673 patients were identified for inclusion. Based on the exclusion criteria, the following groups of patients were excluded: 526 were deceased, 305 patients were under the age of eighteen at the time of treatment, in 59 patients contact information reported in their medical record was not up to date anymore, 25 underwent surgery after second opinion in another institution, and 20 patients did not provide informed consent for participation. Therefore, 738 patients were included for our analyses (Fig. 1). We used the Neer-classification for fracture classification. This classification shows a good inter-observer reliability [24]. X-ray photos of all patients were analyzed with the help of a radiology resident, specializing in trauma radiology.

### Disabilities of the arm, shoulder and hand (DASH)

The DASH questionnaire is a questionnaire that has been designed to be completely filled out by patient without assistance of a physician. It measures the disability of the upper extremity, as well as any symptoms occurring in the preceding week, concerning the health status of a patient. Also, an optional module is added about functionally hazards during work and sports [9, 14]. It has been validated for the proximal part of the upper arm [11] and is also available in Dutch [39]. There are no standardized cut-off points or benchmarks to categorize the DASH score, but cut-off points often used in recent literature are defined as good (no or minimal problems) if <15 points, moderate (problems, still working) if 15–40 points, and poor (problems, not working) if >40 points were obtained [1, 17].

### Health-related quality of life (HrQoL)

The HrQoL was obtained by letting patients fill out the Euro-Qol 5Dimensions (EQ-5D) questionnaire [18, 37]. It is translated and validated into the Dutch language, and also tested on its repeatability, taking age- and sex-adjusted population norms into account [21]. This QoL-questionnaire has, just like the DASH questionnaire, been designed to be filled out completely by patient without any assistance and it includes five questions and an visual analogic self-perception (VAS) scale, ranging from 0 to 100) [38]. Also, the EQ-5D has been proven to show a good internal and external responsiveness for patients suffering from a fracture of the proximal humerus [29].

The EuroQol-utility score (EQ-us) was calculated with the EQ self-classifier. In this article we used the UK EQ-5D index tariff, in absence of a Dutch index tariff, for all health states possible [5–7, 33].

With this calculated index tariff, a single index value can be linked for all possible states of health, where some are hypothetical. Maximum index score is set at 1.00, representing a state of full health. The score indicated by patients on the visual analogic self-perception scale can also be compared and referred with the index tariff.

The reference population we used consisted of a sample of Dutch population, comparable to our cohort (*n* = 4504, UK-tariff): age 50–97 years old (mean = 64, SD = 10), 45% males and 77% low standing diseases like low back pain, asthma and diabetes [13].

### Data analysis

Categorical variables are reported in percentages and frequency, where continuous variables are presented in mean plus/minus standard deviation (±SD) or median and interquartile ranges 25th–75th percentile dependent on distribution. The Mean-Whitney *U* test was used for testing for significance at differences in outcomes between dichotomous characteristics. For nominal patient variables we used the Kruskal–Wallis test. Considered as statistically significant, were *p* values of less than 0.05. Spearman’s rank correlation test was applied to compute associations between the DASH scores and HrQoL.

For conducting all statistical analyses SPSS statistical software 22.0, Chicago, for Microsoft Windows was used.

## Results

### Patients

A total of 410 patients (out of 738) completely filled out and returned the questionnaires, leading to a response rate of 56%. Two-hundred and eighty patients did not respond and 48 patients had to be excluded for returning incomplete (and, therefore, non-usable) questionnaires.

Of all patients included for analyses, 65 were male (16%) and 345 female (84%) with a mean age of 62 ± 12.1 years at the time of fracture and an average follow-up time of 90 ± 48 months. 136 (36%) were 2-part, 158 (40%) 3-part and 97 (25%) were 4-part fractures. 19 X-ray photos were not available anymore at the department of Radiology because of expired medical record retention, so percentages are calculated where 100% is equal to *n* = 391.

Fifty-seven patients (14%) smoked, 33 (8.0%) were diagnosed with Diabetes, and 102 (25.0%) patients reported they were suffering from osteoporosis at the time of the fracture. Patient characteristics are outlined in Table 1.

**Table 1:** Baseline characteristics of the cohort, surviving the follow-up (*n* = 410 patients,) returning the questionnaire completely filled out the DASH and QoL questionnaires

	Total cohort *n* = 410
Age^a^	70 ± 12.0
Age at fracture^a^	62 ± 12.1
Male gender^b^	65 (16%)
Fracture at dominant limb^b^	208 (52%)
Parts of fracture^b,c^	
2-part^d^	136 (36%)
3-part	158 (40%)
4-part	97 (25%)
Months since fracture	89.7 ± 47.9
Smoking^b^	57 (14%)
Diabetes mellitus^b^	33 (8.0%)
Osteoporosis^b,e^	102 (25%)

^a^Mean (±standard deviation) age at time of questionnaire survey

^b^Number of patients (%)

^c^19 X-ray photos were not available anymore at the department of Radiology, so percentages are calculated where 100% is equal to *n* = 391

^d^Avulsion fracture and 2-parts facture were combined

^e^Self-reported

### Functional outcome of the shoulder

The median DASH-score in this cohort was 6.67 [0.83–22.50], after an average follow-up period of 90 ± 48 months. Good scores (<15 points) were obtained in 66%, moderate scores (15–40 points) in 24%, and poor scores (>40 points) in 10%. Table 2 shows the influence of patients’ characteristics on the DASH.

**Table 2:** DASH-score within patient characteristics

	*n*	DASH-score^a^	*p* value^b^
Total cohort	410	6.67 (0.83–22.50)	
Age			
Age ≤65	248	5.00 (0–15.00)	<0.001
Age >65	162	12.5 (3.13–31.67)	
Gender			
Male	65	3.33 (0–12.50)	0.011
Female	345	7.50 (0.83–22.92)	
Dominant arm			
Yes	208	7.50 (0.83–25.21)	0.248
No	202	5.83 (0.83–22.50)	
Parts of fracture			
2-part	136	5.83 (0–15.83)	0.024
3-part	158	6.67 (0.83–19.38)	
4-part	97	14.17 (2.50–32.08)	
Smoking			
Yes	57	10.0 (2.92–34.17)	0.056
No	353	5.83 (0.42–21.67)	
Diabetes			
Yes	33	17.5 (2.50–38.75)	0.018
No	377	6.67 (0.83–20.00)	
Osteoporosis			
Yes	102	10.0 (0.63–31.87)	0.048
No	308	6.67 (0.83–17.50)	

^a^Median (25th–75th percentile)

^b^Statistical comparison for DASH-score within subgroups of patient characteristics

When dividing our cohort in a relatively younger cohort (≤65 years old at the time of fracture, *n* = 248) and an old cohort (*n* = 162), we found significantly different median DASH-scores of 5.0 [0.0–15.0] versus 12.50 [3.13–31.67, *p* < 0.001], respectively.

Multiple regression analysis on our data, showed significant correlations in age (at the time of fracture, *ρ* = 0.293, *p* < 0.001) and the number of fracture parts (*ρ* = 0.143, *p* = 0.005). No significant correlation was seen between number of months since fracture (*ρ* = −0.014, *p* = 0.401).

The optional work-module was filled out completely by 161 patients (39%). Of those, 126 patients reported no problems at all (78%), 25 (16%) reported mild problems, 7 (4%) moderate problems, one patient (<1%) reported severe problems, and only two patients (<1%) were not able to carry out work-related activities anymore due to the fracture. For this (working) subgroup, a median DASH-score of 5.0 [0.0–14.17] was found.

The optional module considering doing sports or playing an instrument was fully completed by 150 (37%) patients. A median DASH-score of 3.75 [0.0–13.75] was found in this subgroup.

At the time of this survey, 169 (41%) patients reported a similar function of the upper extremity compared to the function before fracture [median DASH-score 0.0 (0.0–5.0)]. In 187 (45%) patients, function of the upper arm was slightly reduced with no major disabilities after fracture [median DASH-score 10.83 (5.0–21.88)], and 54 (13%) patients reported major or severe decrease in functionality [median DASH-score 44.58 (30.0–56.04)].

No complaints of pain were documented in 268 (65%) patients and 39 (10%) patients endured pain at least once a month. Only 20 (5%) patients endured pain less than once a day, but less than once a week, and pain was present daily or continuously in 63 (15%) patients. A significant (*p* < 0.001) difference was seen in median DASH score between the group with no pain [2.50 (0.0–8.96)] compared to the group still enduring pain [24.58 (11.67–42.71)].

### Health related quality of life

Mean EQ-us (UK-tariff) in the current cohort was 0.82 ± 0.26. Percentages of impairments reported by patients in the various dimensions of the EQ can be found in Table 3 reported. A mean VAS score of 76.2 ± 16.0 was found.

**Table 3:** EuroQol results

	Overall	Age ≤65	Age >65	*p* value^a^
	*n* = *410*	*n* = *248*	*n* = *162*	
EQ-us (UK-tariff)^b^	0.82 ± 0.26	0.85 ± 0.23	0.78 ± 0.28	0.021
Impairment in dimension^c^				
Mobility (%)	142 (35%)	76 (31%)	66 (41%)	0.038
Self-care (%)	70 (17%)	31 (13%)	39 (24%)	0.004
Usual activity (%)	116 (28%)	60 (24%)	56 (35%)	0.026
Pain/disorder (%)	151 (37%)	88 (36%)	64 (39%)	0.276
Anxiety/depression (%)	49 (12%)	26 (11%)	23 (14%)	0.272
VAS-score^a^	76.2 ± 16.0	77.9 ± 15.1	73.5 ± 16.4	0.006

The outcome scales of the EQ-5D from no problems, mild problems and severe problems were dichotomized to ‘no problems’ versus ‘problems’

^a^Statistical comparison between the group aged less or equal and older than 65 years of age at the time of the fracture

^b^Mean ± SD values

^c^Number of patients (%)

A strong negative correlation was found between DASH-score and the self-reported VAS score, *ρ* = −0.534, *p* = 0.01. An even stronger correlation, *p* = 0.01, was seen between the DASH-score and EQ-US-score, *ρ* = −0.777.

A significant difference was found in the mean EQ-US-score, comparing the younger patients (age ≤65 at the time of the fracture) versus the old patients subgroup (age >65), with a negative effect for the latter group, *p* = 0.021.

When comparing our cohort to the general Dutch population [13], a lower EQ-us was found (difference of 0.02, *p* = 0.083), although not significant. However, if the single dimensions are compared, we do find more impairments reported in all the 5 dimensions, significant in all expect for ‘anxiety/depression’, which is shown in Table 4.

**Table 4:** Quality of life outcome comparison between the Dutch reference population and our study cohort, and subdivided by patients younger or equal to 65 and older than 65 years of age at the time of fracture

	Refer. Pop [13]	Study cohort	*p* value^a^
	*n* = 4504	*n* = 410	
EQ-us^b^	0.84 ± 0.22	0.82 ± 0.26	0.083
Impairment in dimension^c^			
Mobility (%)	29	35	0.01
Self-care (%)	6	17	<0.001
Usual activity (%)	19	28	<0.001
Pain/disorder (%)	39	37	0.424
Anxiety/depression (%)	11	12	0.535

The outcome scales of the EQ-5D from no problems, mild problems and severe problems were dichotomized to ‘no problems’ versus ‘problems’

^a^Statistical comparison between our overall cohort and the reference Dutch population, UK-tariff [39]

^b^Mean ± SD values

^c^Percentage of patients

## Discussion

Aim of this study was to report the functional outcomes and Health related Quality of Life of patients with a proximal (subcapital) fracture of the humerus treated non-operatively. Since current literature lacks long-term follow-up studies, our study yields some important findings which could definitely be used to inform people about the potential functional disabilities they can expect after treatment. Most importantly we found a good median DASH-score (of 6.67) in our cohort, with a long average follow-up time of 90 ± 48 months.

Proximal, and more specifically, subcapital humeral fractures are in the minds of both patients and physicians often associated with an expected decrease in function of the shoulder [25]. Our long-term follow-up study does not necessarily support this assumption. The low median DASH-score in our study indicates that most of patients perceived functional outcomes after a fracture of the proximal humerus are excellent (DASH-score <15 points). Comparison with current literature is difficult because of the absence of similar long follow-up periods. Comparison with pre-fracture scores is obviously not possible, whilst people do not plan the proximal humeral fracture. Other discrepancies between studies might derive from differences in patient characteristics such as gender, age, amount of parts, or the presence of co-morbidities [35].

Comparison can be made with studies examining patients reported shoulder functioning after surgically managed proximal humeral fractures. The comparison especially is interesting in the discussion whether to treat non-operatively or surgically. Current literature mainly consist of short-term studies up to 24 months, reporting post-operative DASH-scores, varying between 16 and 35 points [3, 12, 15, 20, 28, 29, 36]. As mentioned, our study is about long-term functional outcomes and these data are unfortunately also scarce after surgical treatment. However, recently a few long-term follow-up studies have been published, reporting DASH-scores after surgery ranging from 12 to 30 points [2, 11, 27, 30], which are comparable to our results after non-operative treatment. Our study is the first actually reporting these outcomes in a long-term timeline. Also our high response rate of 56%, despite a high average age and the long follow-up period, makes our study valid and of suitable quality.

The reason we preferred DASH questionnaire, to measure functional outcome, over other instruments like the Oxford Shoulder (OSS) and the UCLA shoulder score is that DASH has a very broad scope in functioning of the whole upper extremity. Therewith, limitation of the DASH is that many items may seem irrelevant to patients, especially to patient with a specific condition. Secondly a disadvantage of a specific instruments as the DASH questionnaire is that it does not take into account the history of patients filling out the questionnaire, which can point out lower DASH scores not related to the fracture [29].

Some limitations can be allocated to our study. Obviously, our Study is of a retrospective nature, making it hard to include all consecutive patients since the start of the study, providing some bias in our results. Also, only a post-treatment outcome of the DASH and EQ-5D questionnaire is reported, preventing us to compare the functional and HrQoL outcome with outcomes before the fracture. Rather interesting is the change in (perceived) health, instead of the health at the end of the follow-up period. This would have eliminated bias of other co-morbidities and ageing. Nevertheless, we have added the comparison to an age- and gender matched population to overcome this limitation. Therefore, our outcomes give a good image of what disabilities can be expected after non-operative treatment.

Another limitation lies in the fact that the QoL was only measured at one certain point in time (2015). Because of this, follow-up time of the HrQoL differs from 2 to 16 years, making it hard to interpret these results. If scores were obtained with the same amount of time between fracture and measurement, results were less difficult to clarify.

Thirdly, our overall response rate is 56%. This response is a result of our long follow-up period and a relatively old population (with associated comorbidities like dementia). It is also doubtful whether patients with minimal functional loss will take time to fill out the complete questionnaire.

Although some limitations can be pointed out, useful information on the functional outcomes of proximal humeral fractures after non-operative treatment has been provided.

In conclusion, the long-term functional outcomes, after a non-operatively treated proximal humeral fractures, appears to be good in almost two-third of the patients (66%). Only 10% still suffers from serious impairments or experiences considerable disabilities in daily functioning. Patients with a worse DASH-score also scored lower on the HrQoL score. These outcomes are valuable to inform patients with a proximal humeral fracture about the outcome of non-operative treatment.

## References

[1] Angst F, Goldhahn J, Pap G (2007). Cross-cultural adaptation, reliability and validity of the German Shoulder Pain and Disability Index (SPADI). Rheumatol (Oxf).

[2] Bahrs C, Kühle L, Blumenstock G, Stöckle U, Rolauffs B, Freude T (2015). Which parameters affect medium- to long-term results after angular stable plate fixation for proximal humeral fractures?. J Shoulder Elbow Surg.

[3] Brunner F, Sommer C, Bahrs C (2009). Open reduction and internal fixation of proximal humerus fractures using a proximal humeral locked plate: a prospective multicenter analysis. J Orthop Trauma.

[4] Court-Brown CM, Caesar B (2006). Epidemiology of adult fractures: a review. Injury.

[5] Dolan P (1997). Modeling valuations for EuroQol health states. Med Care.

[6] Dolan P, Gudex C, Kind P (1995) A social tariff for EuroQol: results from an UK general population survey. NHS centre for reviews and dissemination, York. https://www.york.ac.uk/che/pdf/DP138.pdf

[7] Dolan P, Gudex C, Kind P, Williams A (1994) The measurement and valuation of health. Centre for Health Economics, University of York, York

[8] Fjalestad T, Strømsøe K, Blücher J, Tennøe B (2005). Fractures in the proximal humerus: functional outcome and evaluation of 70 patients treated in hospital. Arch Orthop Trauma Surg.

[9] Gummesson C, Atroshi I, Ekdahl C (2003). The disabilities of the arm, shoulder and hand (DASH) outcome questionnaire: longitudinal construct validity and measuring self-rated health change after surgery. BMC Musculoskelet Disord.

[10] Hanson B, Neidenbach P, de Boer P, Stengel D (2009). Functional outcomes after nonoperative management of fractures of the proximal humerus. J Shoulder Elbow Surg.

[11] Hirschmann MT, Fallegger B, Amsler F, Regazzoni P, Gross T (2011). Clinical longer-term results after internal fixation of proximal humerus fractures with a locking compression plate (PHILOS). J Orthop Trauma.

[12] Hirschmann MT, Quarz V, Audigé L (2007). Internal fixation of unstable proximal humerus fractures with an anatomically preshaped interlocking plate: a clinical and radiologic evaluation. J Trauma.

[13] Hoeymans N, van Lindert H, Westert GP (2005). The health status of the Dutch population as assessed by the EQ-6D. Qual Life Res.

[14] Hudak PL, Amadio PC, Bombardier C (1996). Development of an upper extremity outcome measure: the DASH (disabilities of the arm, shoulder and hand) [corrected]. The Upper Extremity Collaborative Group (UECG). Am J Ind Med.

[15] Jones CB, Sietsema DL, Williams DK (2011). Locked plating of proximal humeral fractures: Is function affected by age, time, and fracture patterns. Clin Orthop Relat Res.

[16] Karl JW, Olson PR, Rosenwasser MP (2015). The epidemiology of upper extremity fractures in the United States, 2009. J Orthop Trauma.

[17] Kennedy CA, Beaton DE, Smith P (2013). Measurement properties of the QuickDASH (disabilities of the arm, shoulder and hand) outcome measure and cross-cultural adaptations of the QuickDASH: a systematic review. Qual Life Res.

[18] Krabbe PFM, Stouthard MEA, Essink-Bot ML, Bonsel GJ (1999). The effect of adding a cognitive dimension to the EuroQol multiattribute health-status classification system. J Clin Epidemiol.

[19] Kristiansen B, Barfod G, Bredesen J et al (1987) Epidemiology of proximal humeral fractures. Acta Orthop Scand 58(1):75–77. http://www.ncbi.nlm.nih.gov/pubmed/3577743 10.3109/17453678709146347 3577743

[20] Laflamme GY, Rouleau DM, Berry GK, Beaumont PH, Reindl R, Harvey EJ (2008). Percutaneous humeral plating of fractures of the proximal humerus: results of a prospective multicenter clinical trial. J Orthop Trauma.

[21] Lamers LM, Stalmeier PFM, McDonnell J, Krabbe PFM, Van Busschbach JJG (2005). Kwaliteit van leven meten in economische evaluaties: het Nederlands EQ-5D-tarief. Ned Tijdschr Geneeskd.

[22] Launonen AP, Lepola V, Flinkkilä T (2012). Conservative treatment, plate fixation, or prosthesis for proximal humeral fracture. A prospective randomized study. BMC Musculoskelet Disord.

[23] Lauritzen JB, Schwarz P, Lund B, McNair P, Transbøl I (1993). Changing incidence and residual lifetime risk of common osteoporosis-related fractures. Osteoporos Int.

[24] Mahadeva D, Dias RG, Deshpande SV, Datta A, Dhillon SS, Simons AW (2011). The reliability and reproducibility of the Neer classification system—digital radiography (PACS) improves agreement. Injury.

[25] Maier D, Jaeger M, Izadpanah K, Strohm PC, Suedkamp NP (2014). Proximal humeral fracture treatment in adults. J Bone Joint Surg Am.

[26] Ockert B, Biermann N, Haasters F, Mutschler W, Braunstein V (2013). Reverse shoulder arthroplasty for primary fracture treatment. Displaced three and four part fractures of the proximal humerus in the elderly patient. Unfallchirurg.

[27] Ockert B, Siebenbürger G, Kettler M, Braunstein V, Mutschler W (2014). Long-term functional outcomes (median 10 years) after locked plating for displaced fractures of the proximal humerus. J Shoulder Elbow Surg.

[28] Olerud P, Ahrengart L, Ponzer S, Saving J, Tidermark J (2011). Internal fixation versus nonoperative treatment of displaced 3-part proximal humeral fractures in elderly patients: a randomized controlled trial. J Shoulder Elb Surg.

[29] Olerud P, Tidermark J, Ponzer S, Ahrengart L, Bergström G (2011). Responsiveness of the EQ-5D in patients with proximal humeral fractures. J Shoulder Elbow Surg.

[30] Osterhoff G, Hoch A, Wanner GA, Simmen H-P, Werner CML (2012). Calcar comminution as prognostic factor of clinical outcome after locking plate fixation of proximal humeral fractures. Injury.

[31] Poeze M, Lenssen AF, Van Empel JM, Verbruggen JP (2010). Conservative management of proximal humeral fractures: can poor functional outcome be related to standard transscapular radiographic evaluation?. J Shoulder Elb Surg.

[32] Seeley DG, Browner WS, Nevitt MC, Genant HK, Scott JC, Cummings SR (1991). Which fractures are associated with low appendicular bone mass in elderly women? The Study of Osteoporotic Fractures Research Group. Ann Intern Med.

[33] Shaw JW, Johnson JA, Coons SJ (2005) US valuation of the EQ-5D health states: development and testing of the D1 valuation model. Med Care. 43(3):203–220. http://www.ncbi.nlm.nih.gov/pubmed/15725977. Accessed June 26, 2016 10.1097/00005650-200503000-00003 15725977

[34] Stangl DMR (2015) Proximale Humerusfraktur im fortgeschrittenen Lebensalter: Lebensqualität, klinische Ergebnisse und Institutionalisierung nach primärer inverser Frakturprothesenimplantation. In: *Der Unfallchirurg*. 1–8. doi:10.1007/s00113-015-0009-8 10.1007/s00113-015-0009-8 25986770

[35] Südkamp NP, Audigé L, Lambert S, Hertel R, Konrad G (2011). Path analysis of factors for functional outcome at one year in 463 proximal humeral fractures. J Shoulder Elbow Surg.

[36] Tamimi I, Montesa G, Collado F (2015). Displaced proximal humeral fractures: when is surgery necessary?. Injury.

[37] The_EuroQol_Group (1990). EuroQol–a new facility for the measurement of health-related quality of life The EuroQol Group. Health Policy (New York).

[38] Torrens C, Corrales M, Vilà G, Santana F, Cáceres E (2011). Functional and quality-of-life results of displaced and nondisplaced proximal humeral fractures treated conservatively. J Orthop Trauma.

[39] Veehof MM, Sleegers EJA, van Veldhoven NHMJ, Schuurman AH, van Meeteren NLU (2002). Psychometric qualities of the Dutch language version of the Disabilities of the Arm, Shoulder, and Hand questionnaire (DASH-DLV). J Hand Ther.

